# The intellectual structure and thematic evolution of skin cancer research in the Middle East: A data-driven bibliometric exploration of global integration and emerging fronts (1946–2025)

**DOI:** 10.1097/MD.0000000000050460

**Published:** 2026-08-28

**Authors:** Ahmad Assiri

**Affiliations:** a Department of Dermatology, College of Medicine, Jazan University, Jazan, Saudi Arabia.

**Keywords:** artificial intelligence, bibliometric analysis, collaboration networks, dermatologic oncology, thematic mapping

## Abstract

**Background:**

Skin cancer research in the Middle East has grown substantially over recent decades, yet its intellectual and collaborative landscape remains underexplored. This study aimed to map the evolution, thematic structure, and collaboration patterns of regional skin cancer research (1946–2025), benchmarking these trends against global trajectories to identify emerging conceptual domains and research priorities.

**Methods:**

Six comprehensive Scopus-derived datasets – categorized by duration, document type, and collaboration scope – were analyzed using Bibliometrix (R), VOSviewer, and CiteSpace. Analyses included productivity trends, choropleth mapping, citation impact, comparative metrics, co-authorship networks, keyword co-occurrence, thematic evolution, and cluster detection. Callon centrality–density model was applied to map thematic maturity, and CiteSpace was used to identify emerging clusters (2021–2025). All procedures followed the BIBLIO-checklist.

**Results:**

A total of 179,158 initial records were retrieved from Scopus. After applying progressive filters, 6 datasets were constructed: dataset 1 (n = 7932), dataset 2 (n = 5640), dataset 3 (n = 5329), dataset 4 (n = 3869), dataset 5 (n = 2107), and dataset 6 (n = 1260). Regional research exhibited a 7.38% annual growth rate but lagged in international co-authorship (2.74% vs 33.31% globally). Thematic analyses showed a shift from clinical dermatology toward AI-driven diagnostics, dermoscopy, and melanoma. Turkey, Iran, and Egypt accounted for over 70% of regional output. Visualizations revealed fragmented intra-regional networks but strong external ties with the U.S. and Europe. CiteSpace clustering highlighted melanoma cell lines and absorption spectroscopy as recent focal areas.

**Conclusion:**

Middle Eastern skin cancer research is gradually adopting computational and translational approaches. However, collaboration gaps and limited citation impact underscore the need to strengthen regional networks, promote open access, and invest in integrative, technology-driven research platforms.

## 1. Introduction

Globally, skin cancers rank among the most common malignancies, with 6.6 million cases diagnosed in 2021.^[1,2]^ Incidence has been rising worldwide, especially in regions with high ultraviolet (UV) exposure such as Australasia. In contrast, the Middle East has lower incidence (age-standardized incidence rate [ASR] ~6.7 per 100,000 in 2021),^[3]^ and skin cancers account for only ~2% of cancers in countries like Saudi Arabia.^[4]^ However, skin cancer mortality in the Middle East is rising, reaching ~0.3 per 100,000,^[3]^ reflecting later diagnoses and regional disparities in care.

The regional epidemiological landscape is shaped by significant disparities in demographics, economic development, and healthcare infrastructure. Middle Eastern populations range from 1.6 million in Bahrain to over 118 million in Egypt, with wide-ranging Gross Domestic Products (GDPs) – from Palestine’s USD 13.7 billion to Turkey’s USD 1.57 trillion.^[3,5–7]^ More than 16,000 nonmelanoma skin cancer (NMSC) cases have been reported across the region, with Turkey and Iran contributing nearly 75% of this burden.^[8]^ ASRs vary significantly – from 0.5 per 100,000 in Kuwait to 7.3 in Turkey – averaging around 2.3, which is substantially lower than in many Western countries.^[9,10]^ Mortality is highest in Egypt (1206 deaths) and Turkey (573 deaths), underscoring the influence of population size, registry efficiency, and public health infrastructure.^[11]^ Notably, some high-GDP countries like Qatar, the UAE, and Saudi Arabia report lower incidence rates, likely due to a combination of underreporting, protective clothing norms, and predominantly indoor lifestyles.^[6,7]^ Conversely, countries such as Iran and Lebanon show higher ASRs, which may be attributed to stronger cancer registry systems and higher environmental UV exposure.

Despite the Middle East’s increasing UV exposure risk and growing public health burden from skin cancer, its scientific research contribution to this field remains undercharacterized.^[12]^ Prior bibliometric analyses have assessed general oncology research in the Arab world, Middle East and North Africa (MENA) region, and conflict-affected settings,^[13–16]^ but few have isolated skin cancer as a standalone topic. Existing studies typically aggregate multiple cancer types, which obscures dermatology-specific patterns and regional diagnostic challenges. Furthermore, thematic trends such as artificial intelligence (AI)-driven diagnostics, melanoma-specific translational studies, and regionally relevant public health priorities – like darker skin phenotypes, cultural attitudes toward sun protection, and policy gaps – remain poorly mapped in these reviews. As a result, the extent to which Middle Eastern research addresses region-specific challenges versus reproducing Western paradigms remains unclear.

Recent advances in cancer biology underscore the complex molecular underpinnings of tumor progression and the need for precision oncology approaches. Sirtuins, a family of NAD^+^-dependent deacetylases, have emerged as key modulators of tumor biology across multiple malignancies, including pancreatic and prostate cancers, influencing metabolic reprogramming, immune modulation, and therapy resistance.^[17,18]^ Simultaneously, the role of cancer stem cells has gained prominence, as their ability to self-renew and differentiate underlies tumor initiation, metastasis, and recurrence, emphasizing their relevance across cancer types.^[19]^ Complementing these molecular insights, innovations in immunotherapy, particularly CAR T cell therapy, highlight the importance of selecting optimal tumor-specific antigens to enhance therapeutic efficacy in hematological malignancies.^[20]^ These emerging paradigms in oncology research – ranging from epigenetic regulators to stem cell dynamics and targeted immunotherapies – reflect a broader global shift toward translational and mechanism-based cancer research. However, such translational momentum remains unevenly represented in regional outputs.

This study fills a critical gap by presenting the 1st comprehensive bibliometric analysis of skin cancer research in the Middle East spanning 1946 to 2025. Unlike prior oncology-focused reviews that aggregated broader cancer types or regional outputs, this analysis isolates skin cancer scholarship using 6 Scopus (Elsevier)-derived datasets stratified by document type, time period, and collaboration scope. Leveraging Bibliometrix (University of Naples Federico II), VOSviewer (Leiden University), and CiteSpace (Drexel University), we examine scientific productivity, collaboration networks, citation performance, and thematic evolution. Importantly, we assess conceptual development using Callon centrality–density model and detect molecular experimentation fronts through cluster analysis. This multidimensional framework offers a novel, granular view of dermatologic oncology in the Middle East – yielding actionable insights for policymakers, research funders, and institutions aiming to strengthen regional research capacity, integration, and impact.

## 2. Materials and methods

### 2.1. Data search strategy and Scopus selection

This study implemented a systematic, multitiered search strategy utilizing the Scopus bibliographic database as the primary data source. All the procedures in this study were performed using the BIBLIO-checklist. The selection of Scopus was predicated upon several methodological considerations: its extensive indexing of over 27,000 peer-reviewed journals spanning diverse disciplines, robust citation tracking functionality, standardized metadata architecture facilitating institutional and authorial disambiguation, and native compatibility with established bibliometric analysis software including Bibliometrix, VOSviewer, and CiteSpace.

The search protocol implemented structured Boolean operators (File S1, Supplemental Digital Content 1) incorporating a comprehensive dermatological oncology lexicon encompassing 9 key terms: “skin cancer,” “cutaneous cancer,” “cutaneous malignancy,” “skin carcinoma,” “skin malignancy,” “cutaneous neoplasm,” “cutaneous neoplasms,” “skin neoplasm,” and “skin neoplasms.” These terms were queried within title–abstract–keywords fields and systematically cross-referenced with institutional affiliation filters representing 17 Middle Eastern countries, ensuring geographic specificity while maintaining search sensitivity. In this study, the term “Middle Eastern countries” refers to the following 17 nations based on geographic, cultural, and bibliometric conventions: Bahrain, Cyprus, Egypt, Iran, Iraq, Israel, Jordan, Kuwait, Lebanon, Oman, Palestine, Qatar, Saudi Arabia, Syria, Turkey, United Arab Emirates, and Yemen. This regional classification aligns with definitions commonly used in international bibliometric analyses and institutional frameworks such as those of the WHO Eastern Mediterranean Region and the United Nations.

**Figure 1.**
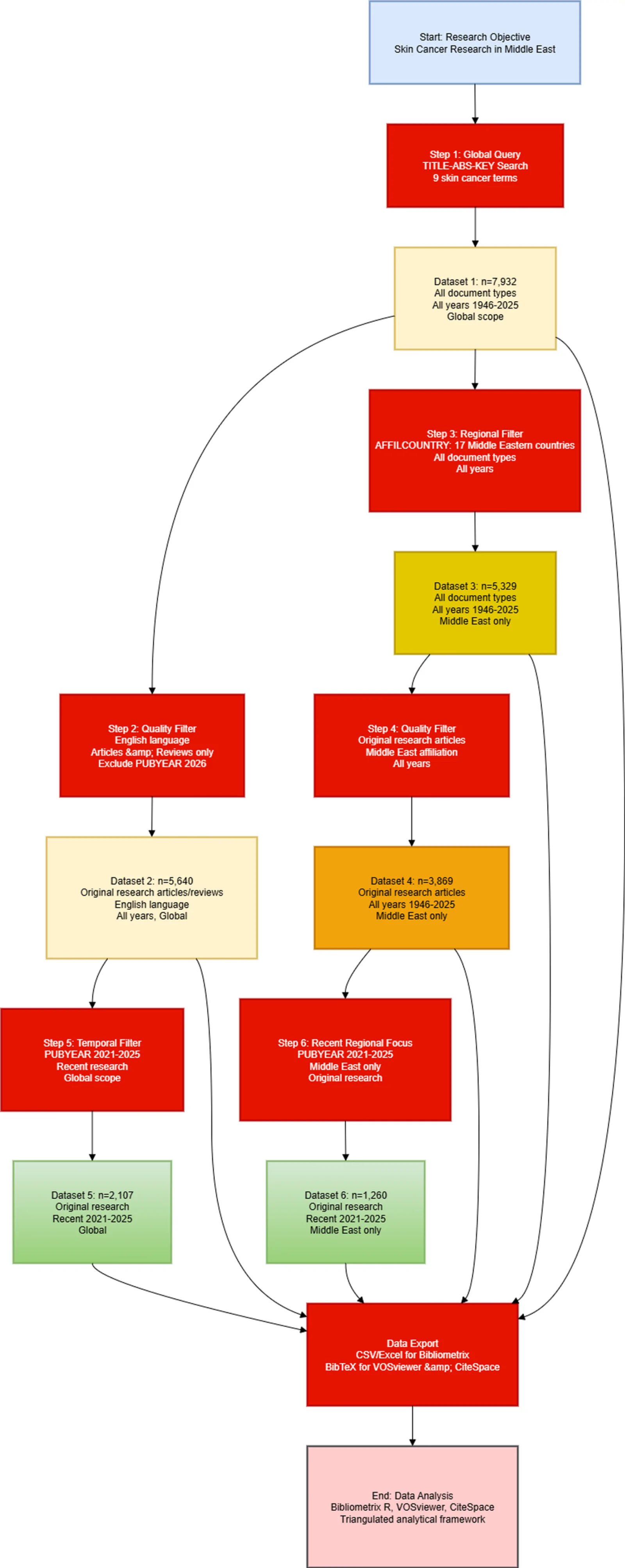
Systematic search strategy and dataset stratification workflow for bibliometric analysis of skin cancer research in Middle Eastern countries (1946–2025). The flowchart delineates 6 progressive datasets derived through sequential application of quality, geographical, and temporal filters within the Scopus database. Color-coding distinguishes baseline global datasets (yellow), regional Middle Eastern subsets (red), and contemporary research corpora (green). Sequential refinement steps illustrate the methodological framework enabling comprehensive triangulated analysis through Bibliometrix, VOSviewer, and CiteSpace platforms. All the procedures in this study were performed using the BIBLIO-checklist.

The extraction procedure (Fig. 1) produced 6 stratified datasets through progressive refinement, each tailored to a specific analytical aim. Dataset 1 (n = 7932) established a global baseline by including all-document types across all years (1946–2025) without geographic limitation. Dataset 2 (n = 5640) applied quality filters to retain only English-language original research articles and reviews globally, excluding gray literature. Dataset 3 (n = 5329) isolated all skin cancer-related documents affiliated with Middle Eastern institutions across the full time span. Dataset 4 (n = 3869) further refined this subset by including only original research articles from Middle Eastern countries, thereby enhancing methodological consistency. Dataset 5 (n = 2107) captured recent global original research (2021–2025) to enable benchmarking of emerging trends. Dataset 6 (n = 1260) focused exclusively on recent (2021–2025) original research from Middle Eastern institutions to assess current regional output. This 6-tiered framework enabled robust comparative analysis across geographic, temporal, and methodological axes, offering a triangulated perspective on the evolution, productivity, and thematic maturation of skin cancer research in the Middle East.

Each of the 6 datasets was defined by mutually exclusive filters – year range, collaboration scope, and document type – to allow comparative benchmarking. Overlap between datasets was avoided by design.

### 2.2. Inclusion and exclusion criteria

This study included Scopus-indexed documents from 1946 to 2025 containing at least 1 of 9 predefined skin cancer terms in the title, abstract, or keywords. Only English-language original research articles and reviews were retained, excluding gray literature and publications beyond 2025. Regional datasets were filtered for 17 Middle Eastern countries using institutional affiliation. Temporal segmentation isolated recent outputs (2021–2025). Exclusion criteria involved non-English texts, non-research formats (e.g., editorials and letters), and future-dated records (e.g., PUBYEAR 2026). The 2025 dataset includes only early-access publications already indexed in Scopus as of the final search date (May 2025), ensuring data accuracy and consistency.

### 2.3. Analytical approach

To systematically analyze the structure, performance, evolution, and conceptual landscape of skin cancer research in the Middle East, a multifaceted bibliometric methodology was employed, integrating quantitative, geospatial, network-based, and content-centric analyses. Three specialized bibliometric tools were used throughout: Bibliometrix (R package), VOSviewer (version 1.6.20), and CiteSpace (version 6.2.R2).^[21–23]^ Each tool offered complementary functionalities across dataset configurations, allowing triangulated insights into scientific productivity, collaboration, impact, and thematic evolution. In Section 3.2, the primary bibliometric indicators – such as total publications, sources, author productivity, international collaboration rates, and citation impact – were computed using Bibliometrix.^[21]^ Comparative evaluations were conducted across 4 Scopus-derived datasets, stratified by geographic scope (global vs Middle East), study period (1946–2025 vs 2021–2025), and document type (all documents vs original articles). These indicators were synthesized to understand longitudinal trends and regional disparities in skin cancer scholarship.

Section 3.3 integrated bibliometric data with epidemiological and economic statistics to explore the relationship between national GDP and NMSC incidence across 17 Middle Eastern countries. GDP figures and cancer incidence rates were visualized using bar and line plots to contextualize research activity within broader socioeconomic and disease burden frameworks. Temporal publication dynamics (Section 3.4) were examined by plotting annual research output across 6 dataset configurations. Trends were visualized using Bibliometrix’s publication trend functions, enabling identification of inflection points and growth phases from 1946 to 2025. Section 3.5 used choropleth mapping to assess the geospatial distribution of skin cancer research output across Middle Eastern countries. This was performed using Bibliometrix’s mapping capabilities and refined through Excel-based visual encodings to depict historical, filtered, and recent publication densities.

Citation impact analysis (Section 3.6) utilized VOSviewer to create density visualizations, quantifying country-level citation frequencies and co-citation clusters.^[23]^ These maps were generated using full-count methods and normalized citation link strengths to identify influential countries and citation hotspots. Four panels were produced to distinguish historical versus recent data and global versus regional collaboration dynamics. International collaboration networks (Section 3.7) were modeled using VOSviewer’s network visualization feature, which generated co-authorship networks based on country affiliations. Link strength, node size, and spatial distribution represented collaborative intensity and integration within global or regional scholarly ecosystems. In Section 3.8, authorship patterns were extracted using Bibliometrix’s biblioAnalysis() function. Top contributors were ranked by total publications across 6 dataset configurations, revealing authorship dominance and temporal shifts in regional scholarly leadership.

Journal distribution analysis (Section 3.9) used the same function to identify top publication outlets, ranked by total documents and filtered by article type and time period. This revealed evolving dissemination strategies and platform preferences.

Thematic keyword co-occurrence patterns (Section 3.10) were explored using VOSviewer, employing author keyword networks. Nodes represented terms, links indicated co-occurrence, and clustering was based on LinLog/modularity.^[23]^ Four visualizations revealed thematic density, cluster count, and term frequency across temporal and geographic partitions.

Section 3.11 employed Bibliometrix’s thematic mapping using Callon centrality–density model to classify themes as motor, basic, niche, or emerging/declining. Co-word matrices were constructed from author keywords, and cluster placement on the thematic map provided structural insights into thematic maturity and relevance.^[21]^ The co-word matrix parameters were as follows: a minimum keyword frequency threshold of 5 and normalization using the association strength method, enhancing the reproducibility of the thematic maps. Overlay visualization (Section 3.12) was conducted in VOSviewer, leveraging temporal co-word overlays to identify emerging research fronts based on average keyword publication year and frequency. Color gradients (blue to yellow) denoted the age of topic emergence. Finally, Section 3.13 used CiteSpace for clustering analysis based on co-cited keywords within the 2021 to 2025 Middle Eastern dataset. Clustering algorithms computed modularity (*Q*) and silhouette values to assess structural integrity. Each cluster was labeled via log-likelihood ratio and analyzed for topical focus (e.g., melanoma, thermodynamics, and toxicology). Together, these multi-tool, multilayered analyses offered a robust and nuanced examination of the Middle Eastern skin cancer research landscape over time.

## 3. Results

### 3.1. Key results summary

This study revealed several critical insights into the landscape of skin cancer research in the Middle East (1946–2025):

1. Growth trend: Regional publication output exhibited a sustained annual growth rate of 7.38%, indicating increasing research engagement across the decades.
2. Collaboration gap: International co-authorship remains markedly limited in the Middle East (2.74%) compared to global benchmarks (33.31%), with fragmented intra-regional networks.
3. Leading contributors: Turkey, Iran, and Egypt accounted for over 70% of the region’s total output, with Turkey consistently ranking highest across all metrics.
4. Thematic evolution: Thematic mapping and clustering analyses revealed a shift from traditional clinical dermatology toward emerging areas such as AI-assisted diagnostics, melanoma research, and translational oncology.
5. Recent fronts: CiteSpace analysis (2021–2025) identified novel research clusters including melanoma cell line experimentation and absorption spectroscopy, suggesting growing interest in molecular and photochemical methodologies.

These findings contextualize the detailed results presented in the subsequent subsections.

### 3.2. Main bibliometric indicators and dataset characteristics

Comprehensive bibliometric analysis across 4 dataset configurations (Table 1) reveals substantial variations in research productivity, collaboration patterns, and citation impact, highlighting critical disparities between globally collaborative and regionally focused skin cancer research (Fig. 8). The global collaborative dataset spanning 1946 to 2025 encompasses 5640 documents published in 1662 unique sources by 19,761 authors, demonstrating a robust annual growth rate of 7.97%. Citation metrics indicate strong scholarly impact with an average of 24.98 citations per document, nearly double the performance of Middle Eastern-focused research. International co-authorship reached 33.31%, reflecting the highly collaborative nature of global skin cancer scholarship, with an average of 5.2 coauthors per document. In contrast, Middle Eastern-focused research (1946–2025) comprises 3868 documents from 1234 sources contributed by 11,599 authors, with a 7.38% annual growth rate. However, citation performance remains substantially lower at 13.18 citations per document – representing only 52.8% of global collaborative research impact. Most critically, the international co-authorship rate stands at merely 2.74%, indicating predominantly isolated national or institutional research efforts with minimal cross-border collaboration. This represents a 12-fold difference compared to global collaborative patterns, constituting a fundamental structural weakness constraining knowledge exchange, methodological advancement, and research visibility (Table 1).

**Table 1:** Main bibliometric indicators and dataset characteristics.

Characteristics	All documents		Original articles	
	Global + ME	ME	Global + ME	ME
Timespan	1946: 2025	1946: 2025	2021: 2025	2021: 2025
Sources	1662	1234	926	626
Documents	5640	3869	2107	1260
Annual growth rate %	7.97	7.38	2.42	5.45
Document average age	10.7	12.2	1.94	1.91
Average citations per doc	24.98	13.18	12.02	6.752
Keywords plus (ID)	23483	16666	13959	9461
Author’s keywords (DE)	30,162	6407	17,620	3257
Authors	19,761	11,599	9179	4686
Authors of single-authored docs	200	197	66	65
Single-authored docs	238	233	76	75
Co-authors per doc	5.2	4.5	5.53	4.62
International co-authorships %	33.31	2.744	44.59	5.957

ME = Middle East.

**Figure 2.**
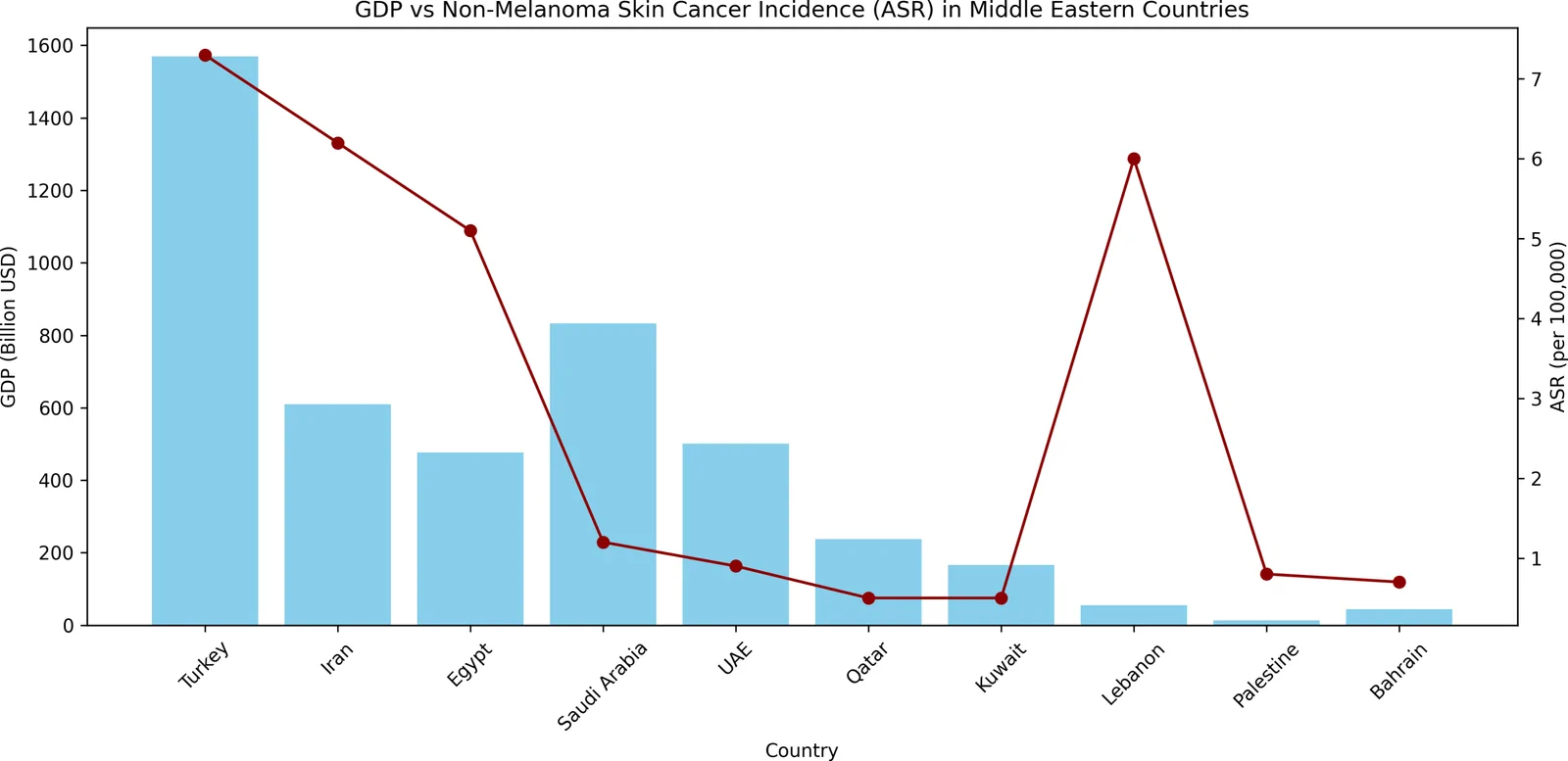
Relationship between national GDP and nonmelanoma skin cancer incidence across Middle Eastern countries (2022–2025). The gray bars represent total GDP (in million USD), while the red line indicates the age-standardized incidence rate (ASR) of nonmelanoma skin cancer per 100,000 population. The plot reveals notable disparities: higher-income countries such as Saudi Arabia, the UAE, and Qatar show relatively low reported incidence rates, while middle-income nations like Turkey and Iran report higher ASRs – suggesting potential under-reporting or differences in diagnostic infrastructure and registry completeness across the region. GDP = Gross Domestic Product.

**Figure 3.**
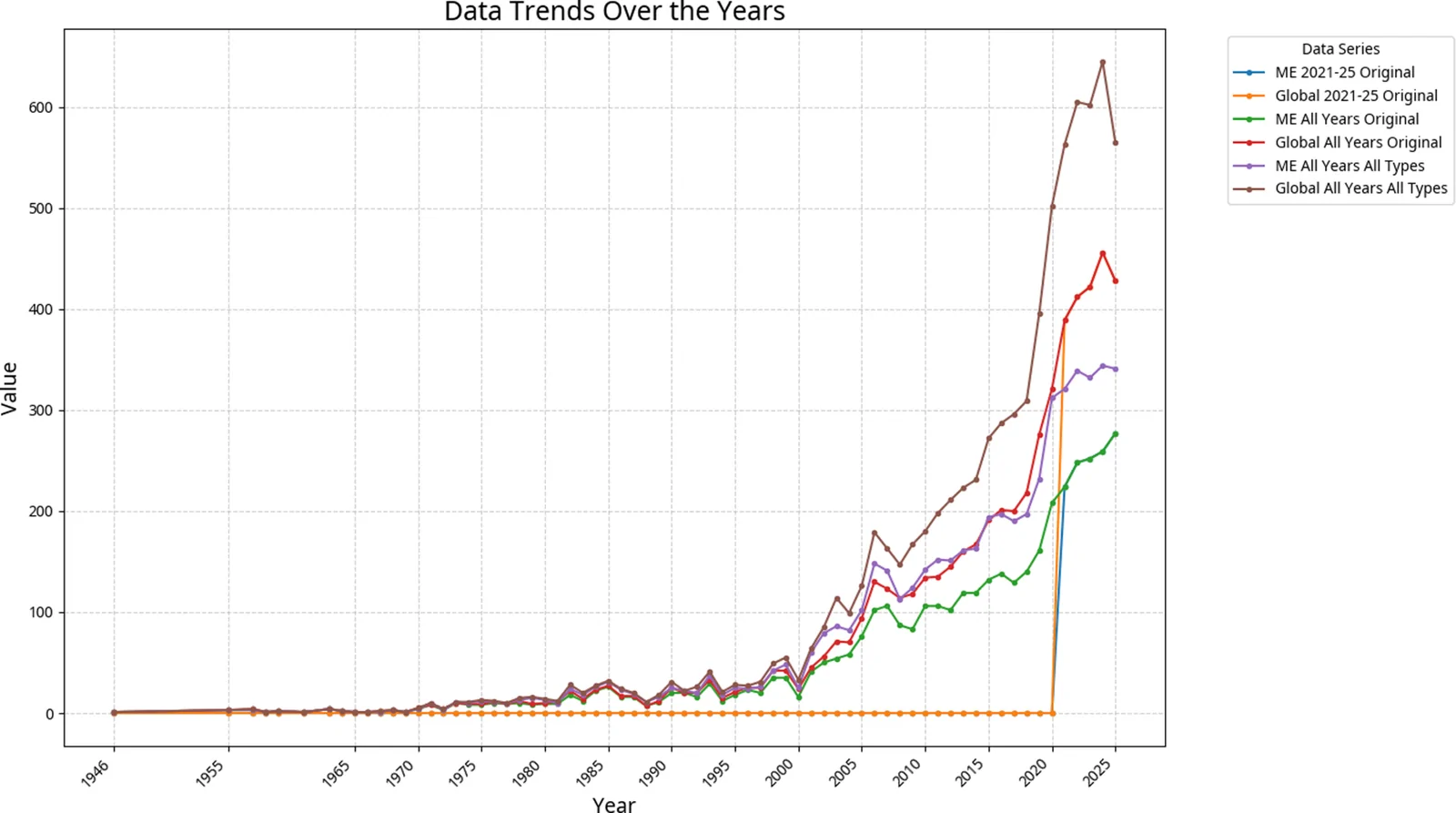
Comparative trends in skin cancer research publications in Middle Eastern and global contexts (1946–2025). The figure illustrates annual publication counts across 6 datasets extracted from Scopus, categorized by region (Middle East vs global), year range, and document type. While global research output shows a steep rise after 2000, Middle Eastern publications also exhibit steady growth, particularly after 2015, reflecting expanding regional engagement in skin cancer research and international collaboration.

**Figure 4.**
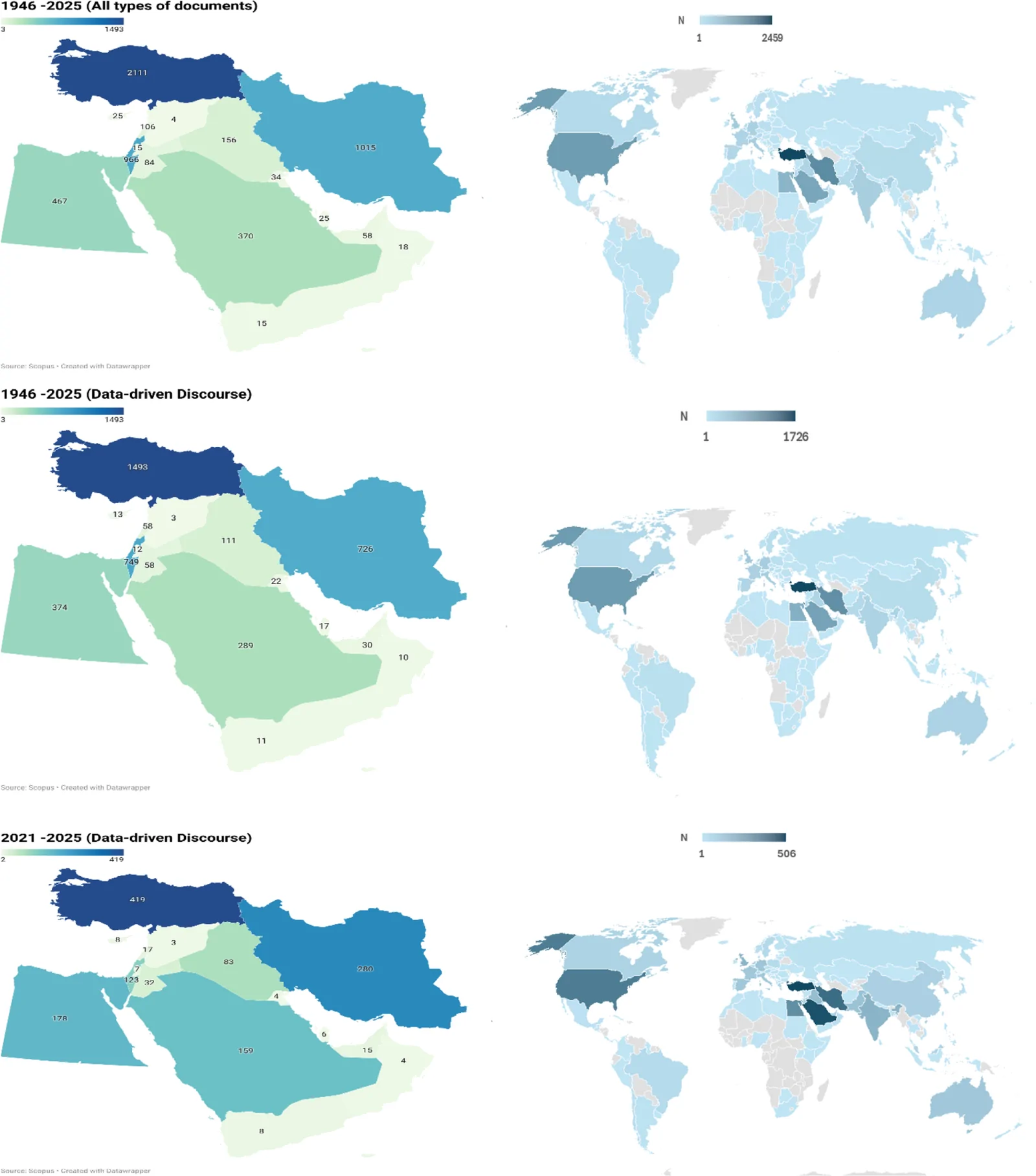
Publication trends in skin cancer research across 6 dataset configurations. The comparison illustrates temporal patterns between Middle Eastern and global research output, stratified by publication timeframe (recent 2021–2025 vs historical), document type (original research vs all types), and geographic scope. Notable growth trajectories are observed in both regional and global datasets, with distinct publication patterns emerging in recent years, reflecting increased research activity and diversification of document types in skin cancer literature.

**Figure 5.**
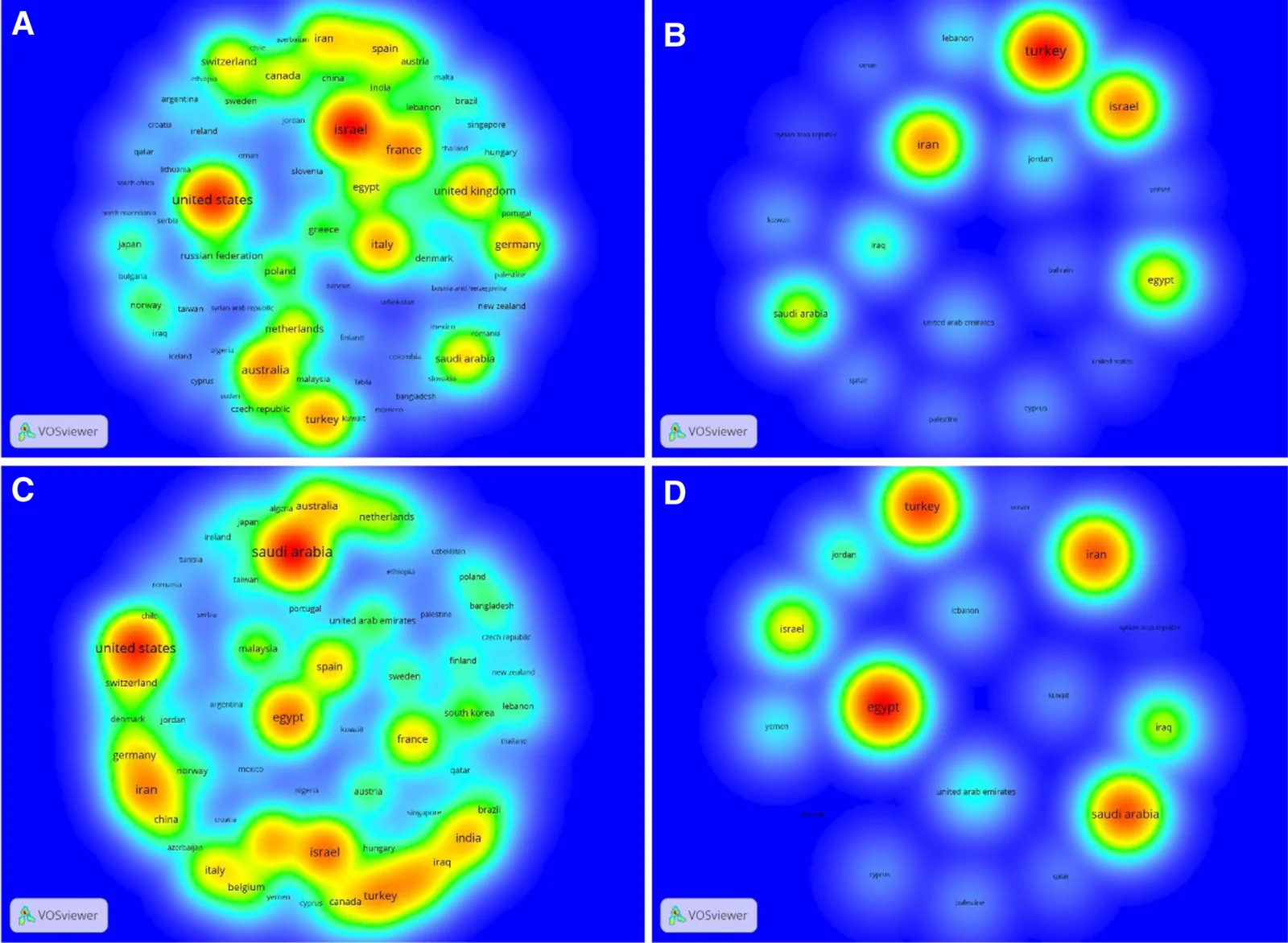
Density visualization of country-level citation impact in skin cancer research (VOSviewer analysis). (A) Global collaborative original research (5640 documents); (B) Middle Eastern-focused original research (3869 documents); (C) recent global collaborative research (2021–2025, 2107 documents); and (D) recent Middle Eastern-focused research (2021–2025, 1260 documents). Heat map intensity indicates citation density, with red representing the highest impact and blue the lowest. Node size reflects document volume; spatial proximity indicates co-citation patterns and collaborative networks.

**Figure 6.**
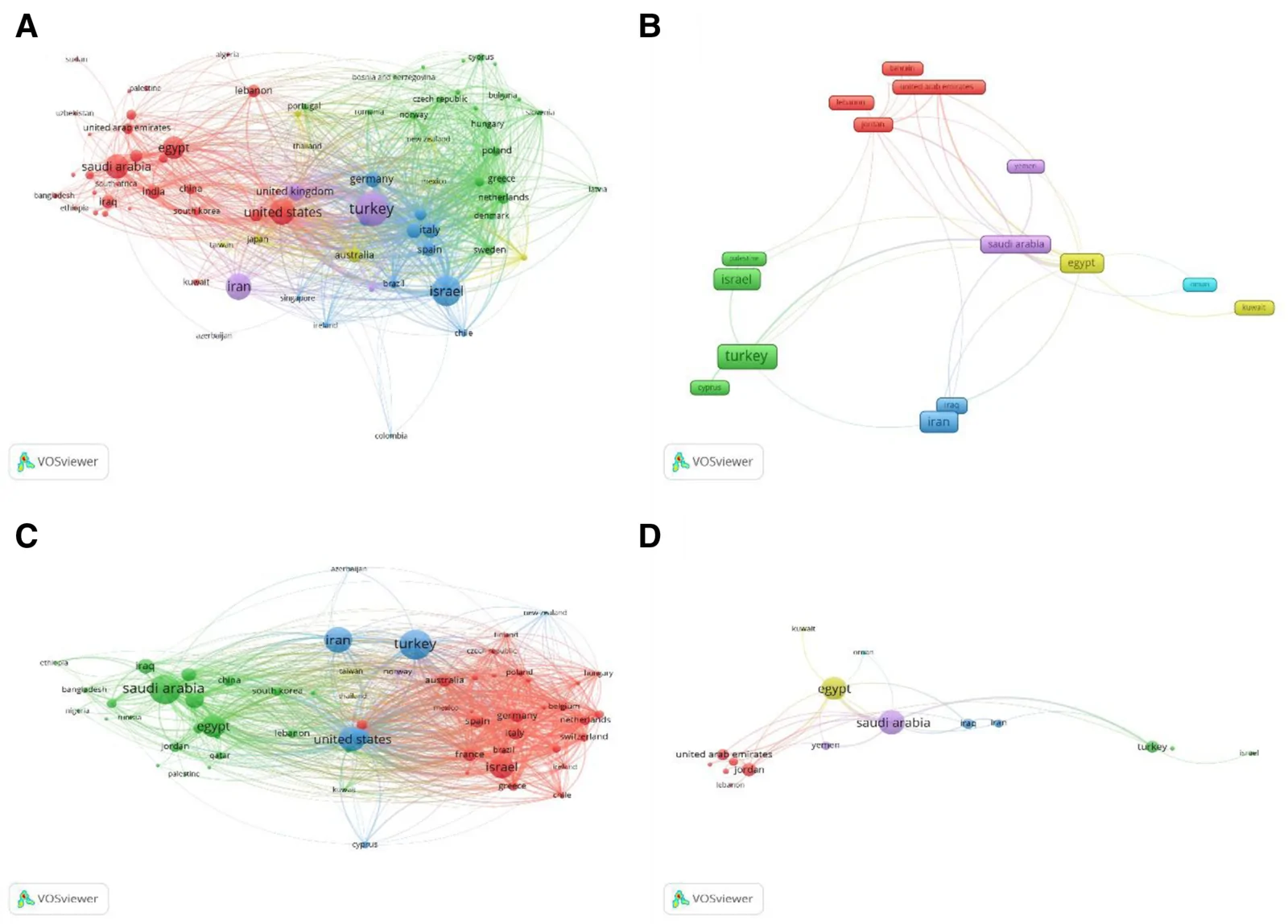
Network visualization of international collaboration patterns in skin cancer research (VOSviewer analysis). (A) Global collaborative original research network (5640 documents); (B) Middle Eastern-focused research network (3869 documents); (C) recent global collaborative network (2021–2025, 2107 documents); and (D) recent Middle Eastern-focused network (2021–2025, 1260 documents). Node size represents document volume; connecting lines indicate collaborative relationships; line thickness reflects collaboration intensity; node colors denote distinct research clusters. Total link strength quantifies the cumulative intensity of a country’s collaborative connections within the network, with higher values indicating greater integration and partnership engagement.

**Figure 7.**
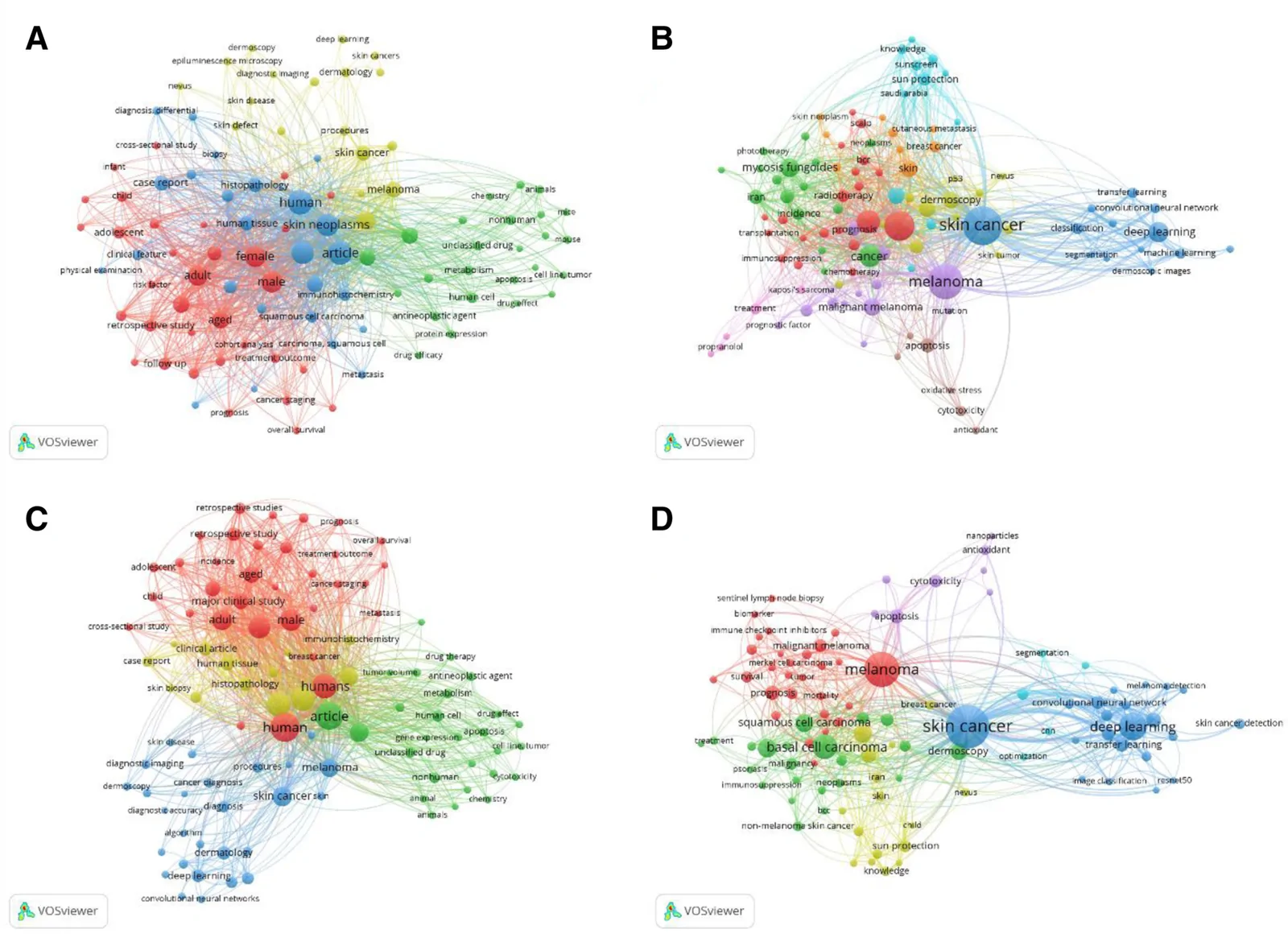
Network visualization of author keyword co-occurrence patterns in skin cancer research (VOSviewer analysis). (A) Global collaborative original research (5640 documents, 4 clusters, 4845 links); (B) Middle Eastern-focused original research (3869 documents, 9 clusters, 915 links); (C) recent global collaborative research (2021–2025, 2107 documents, 4 clusters, 4450 links); and (D): recent Middle Eastern-focused research (2021–2025, 1260 documents, 6 clusters, 618 links). Node size indicates keyword frequency; connecting lines represent co-occurrence within publications; line thickness reflects co-occurrence strength. Distinct color clusters identify thematically related research domains, with spatial proximity indicating conceptual similarity and collaborative keyword usage patterns across the literature.

Recent datasets (2021–2025) demonstrate evolving dynamics. Global collaborative research includes 2106 documents with 12.02 citations per document despite recent publication, while Middle Eastern research encompasses 1259 documents averaging 6.75 citations per document. Encouragingly, Middle Eastern research exhibits higher recent growth (5.45% annually) compared to global research (2.42%), suggesting accelerating regional activity. International co-authorship improved to 5.96% in recent Middle Eastern research – more than doubling historical rates but remaining far below the global benchmark of 44.59%. Co-authorship patterns show 5.53 authors per document in recent global research versus 4.62 in Middle Eastern research, indicating smaller, less interdisciplinary research teams regionally. These metrics (Table 1) collectively underscore persistent gaps in collaborative infrastructure, international integration, and research impact requiring systematic intervention.

### 3.3. National GDP and skin cancer incidence

Figure 2 explores the relationship between national GDP (in millions USD) and NMSC incidence across Middle Eastern countries. Populations range widely – from Bahrain (1.6 million) to Egypt (118 million) – mirroring substantial economic disparities (e.g., Palestine: USD 13,700 million vs Turkey: USD 1570,000 million). Reported NMSC cases exceed 16,000, primarily from Turkey (8229) and Iran (4077), together accounting for nearly 75% of the regional total. ASRs vary from 0.5 per 100,000 in Kuwait to 7.3 in Turkey, averaging ~2.3 – much lower than in Western regions. Mortality is highest in Egypt (1206) and Turkey (573), reflecting both population size and registry robustness. Wealthier Gulf states like Qatar and Saudi Arabia report low incidence despite high GDPs – possibly due to indoor lifestyles, cultural clothing, and underreporting. Middle-income countries (e.g., Iran and Turkey) exhibit higher ASRs, suggesting stronger diagnostic capacity and greater UV exposure. These findings reveal marked heterogeneity in surveillance infrastructure, underscoring the need for improved cancer registries and harmonized reporting systems.

### 3.4. Temporal distribution of skin cancer research

Figure 3 illustrates a steady rise in publication volume from 1946 to 2025. From sparse activity before 1980, output gradually increased through the 1980s and 1990s. A sharp growth trajectory began in the 2000s, with Middle Eastern original research rising from 16 papers (2000) to 208 (2020). All-document types reached 321 in 2020. A similar trend was observed globally (502 papers in 2020). Between 2021 and 2025, Middle Eastern original research averaged 252 annual publications, while global output averaged 421. The gap between total and original research widened in recent years, indicating increased conference and gray literature. Partial 2025 data (277 regional vs 428 global papers) suggest continued strong activity. These patterns demonstrate the region’s growing scholarly engagement, particularly in the last 2 decades.

### 3.5. Geospatial distribution of skin cancer research publications in Middle Eastern countries

Figure 4 presents publication volume by country across 3 configurations. Historically (1946–2025, all types), Turkey leads with 2111 publications, followed by Iran (1015) and Egypt (467). Saudi Arabia shows moderate output (370), while nations like Yemen (15) and Bahrain (4) lag behind. When filtered for peer-reviewed articles (dataset 4), Turkey (1493) and Iran (726) maintain their lead, highlighting methodological rigor. In the recent period (2021–2025, dataset 6), Iran slightly surpasses Turkey (420 vs 419), signaling an emerging shift in research leadership. Egypt ranks 3rd (173), while Gulf states show modest gains. These maps highlight persistent disparities in research infrastructure and regional capacity, with Turkey, Iran, and Egypt accounting for the majority of output. The findings suggest that research productivity reflects national investments in healthcare, epidemiologic burden, and institutional maturity.

### 3.6. Citation impact

Density visualization analysis using VOSviewer reveals substantial heterogeneity in citation impact across countries contributing to skin cancer research, with marked distinctions between globally collaborative and Middle East-focused datasets (Fig. 5). Figure 5A, representing original research with global collaboration, demonstrates the United States, Australia, Israel, and European nations as predominant citation hubs. Among Middle Eastern countries, Israel, Saudi Arabia, Turkey, and Egypt emerge as high-density nodes, suggesting significant citation impact and integration within international research networks. Figure 5B, depicting Middle Eastern research without global collaboration, presents regional concentration, with Turkey demonstrating the highest citation density, followed by Israel, Iran, and Egypt. Quantitative examination reveals Israel’s exceptional citation efficiency with 56,151 citations from 1237 documents (45.4 citations per document globally), substantially exceeding regional counterparts. The United States achieved 47,339 citations from 815 documents (58.1 per document), representing the highest per-document impact among major contributors. Recent publications (2021–2025; Fig. 5C and D) demonstrate evolving patterns, with persistent dominance of established research powerhouses alongside emerging Middle Eastern contributors. Turkey maintained regional leadership with 1465 historical documents generating 15,643 citations, though per-document citation rates (10.7) remained modest compared to Israel (18.5) and Egypt (18.5). The visualization effectively captures not only absolute citation volume but also conceptual clustering and interconnectedness of contributing nations, illustrating the citation premium associated with global collaboration (Fig. 5).

### 3.7. International collaboration networks and research partnerships

Network visualization analysis reveals distinct collaboration patterns between globally integrated and regionally focused skin cancer research (Fig. 6). Figure 6A, representing global collaborative research (5640 documents), demonstrates a highly interconnected network dominated by established research powers. The United States exhibits the strongest collaborative linkages (total link strength: 2549), functioning as the primary hub within international research networks. Israel maintains robust connectivity (link strength: 1854), followed by Italy (1728), the United Kingdom (1580), France (1556), Germany (1517), Spain (1499), and Australia (1341). This configuration reflects well-established North–South collaborative frameworks, wherein high-income nations with mature research infrastructure serve as anchor institutions for multinational partnerships.

Middle Eastern nations demonstrate variable integration within these global networks. Among regional countries, Saudi Arabia, Egypt, and Turkey emerge as principal collaborative nodes, though their link strengths remain substantially lower than Western counterparts. Figure 6C, depicting recent global collaborations (2021–2025, 2107 documents), shows the United States maintaining predominance (link strength: 1220), while regional actors including Saudi Arabia (694), Israel (790), Spain (775), the United Kingdom (769), Italy (739), and Germany (703) sustain intensive collaborative activities.

Figure 6B, illustrating Middle Eastern research without global collaboration (3869 documents), presents a fundamentally different network topology characterized by limited interconnectivity. Saudi Arabia emerges as the regional collaborative leader (link strength: 76), followed by Egypt (74), Turkey (24), Jordan (19), United Arab Emirates (19), Iraq (11), Israel (11), and Qatar (8). These modest link strengths indicate predominantly isolated research activities with minimal cross-border partnerships within the region itself. Figure 6D (recent Middle Eastern-focused research, 1260 documents) demonstrates Saudi Arabia (60), Egypt (52), Jordan (17), Turkey (14), United Arab Emirates (12), Iraq (11), Iran (7), and Qatar (7) as primary regional collaborators, suggesting nascent but growing intra-regional research networks.

The stark contrast between globally collaborative and regionally isolated network configurations underscores critical infrastructure and partnership gaps. While Middle Eastern institutions increasingly engage with international research communities – particularly through partnerships with United States and European institutions – intra-regional collaboration remains underdeveloped. This pattern may reflect linguistic barriers, competitive rather than collaborative institutional cultures, limited regional funding mechanisms supporting multinational consortia, and historical emphasis on North–South rather than South–South partnerships. The visualization reveals opportunities for strengthening regional research capacity through enhanced intra-Middle Eastern collaborative frameworks, potentially leveraging established institutions in Saudi Arabia, Egypt, and Israel as regional coordination hubs to facilitate knowledge exchange and capacity building across less-resourced nations within the region (Fig. 6).

### 3.8. Authorship patterns

Analysis of authorship patterns reveals significant variations across dataset configurations, reflecting the scope and collaborative nature of skin cancer research in the Middle East (Table 2). Among all documents with global collaboration, Scope, A. emerges as the most prolific author with 93 publications, followed by Hodak, E. (78 publications) and Schachter, J. (70 publications). When filtered to original research articles, the ranking shifts, with Scope, A. maintaining leadership (66 publications), closely followed by Hodak, E. (65 publications) and Tas, F. (63 publications). The recent period (2021–2025) demonstrates evolving authorship prominence, with Akay, B.N. leading original research output (23 publications), followed by Hodak, E. (22 publications) and Tas, F. (19 publications). In Middle Eastern-focused research without global collaboration, Tas, F. emerges as the dominant contributor (69 all documents, 63 original research), with Hodak, E. and Erturk, K. also demonstrating substantial productivity. Recent Middle Eastern research (2021–2025) shows Tas, F., Erturk, K., and Shalom, A. as primary contributors (Table 2). This authorship distribution suggests concentrated research expertise within specific institutions and potential opportunities for expanding the investigator base across the region.

**Table 2:** Authorship patterns in Middle Eastern skin cancer research.

Middle Eastern skin cancer research with global collaboration						Middle Eastern skin cancer research					
All documents		Original research		Original research (2021–2025)		All documents		Original research		Original research (2021–2025)	
Author	N	Author	N	Author	N	Author	N	Author	N	Author	N
Scope, A.	93	Scope, A.	66	Akay, B.N.	23	Tas, F.	69	Tas, F.	63	Tas, F.	19
Hodak, E.	78	Hodak, E.	65	Hodak, E.	22	Hodak, E.	49	Hodak, E.	40	Erturk, K.	16
Schachter, J.	70	Tas, F.	63	Tas, F.	19	Demirkesen, C.	47	Erturk, K.	38	Shalom, A.	14
Tas, F.	69	Schachter, J.	62	Khan, M.A.	18	Baykal, C.	41	Feinmesser, M.	33	Baykal, C.	13
Akay, B.N.	64	Akay, B.N.	43	Scope, A.	17	Erturk, K.	41	David, M.	30	Berl, A.	13
Demirkesen, C.	49	Puig, S.	40	Erturk, K.	16	Akay, B.N.	37	Demirkesen, C.	27	Akay, B.N.	12
Puig, S.	45	David, M.	38	Shalom, A.	16	David, M.	35	Schachter, J.	27	Osanloo, M.	11
David, M.	43	Erturk, K.	38	Berl, A.	15	Feinmesser, M.	35	Akay, B.N.	24	Hodak, E.	10
Baykal, C.	42	Feinmesser, M.	38	Marghoob, A.A.	15	Schachter, J.	31	Barzilai, A.	23	Barzilai, A.	8
Erturk, K.	41	Azizi, E.	32	Baykal, C.	14	Barzilai, A.	26	Baykal, C.	21	Karaarslan, I.	7

### 3.9. Journal distribution and publication outlets

The distribution of publications across journals demonstrates distinct patterns between globally collaborative and Middle East-focused research (Table 3). Among all documents with global collaboration, the International Journal of Dermatology predominates with 188 publications, followed by the Journal of the European Academy of Dermatology and Venereology (151 publications) and the Journal of the American Academy of Dermatology (124 publications). When restricted to original research articles, these journals maintain prominence, with 125, 97, and 89 publications respectively. Recent publications (2021–2025) reveal Scientific Reports as the leading outlet (52 articles), followed by Diagnostics (41 articles) and the Journal of Cosmetic Dermatology (27 articles), suggesting a shift toward open-access and multidisciplinary platforms. Middle Eastern research without global collaboration exhibits different journal preferences, with the International Journal of Dermatology (150 all documents, 103 original research) and Dermatology (83 all documents, 71 original research) serving as primary venues (Table 3). Recent Middle Eastern-focused research favors Scientific Reports (32 articles) and the Journal of Cosmetic Dermatology (24 articles). This distribution pattern indicates evolving publication strategies, with increasing preference for high-visibility, open-access journals that enhance research dissemination and citation potential across the region.

**Table 3:** Journal distribution and publication outlets.

Middle Eastern skin cancer research with global collaboration						Middle Eastern skin cancer research					
All documents		Original research		Original research (2021–2025)		All documents		Original research		Original research (2021–2025)	
Source	N	Source	N	Source	N	Source	N	Source	N	Source	N
International Journal of Dermatology	188	International Journal of Dermatology	125	Scientific Reports	52	International Journal of Dermatology	150	International Journal of Dermatology	103	Scientific Reports	32
Journal of the European Academy of Dermatology and Venereology	151	Journal of the American Academy of Dermatology	97	Diagnostics	41	Journal of the European Academy of Dermatology and Venereology	97	Dermatologic Surgery	71	Journal of Cosmetic Dermatology	24
Journal of the American Academy of Dermatology	124	American Journal of Dermatopathology	89	Journal of Cosmetic Dermatology	27	Dermatologic Surgery	83	American Journal of Dermatopathology	59	Archives of Dermatological Research	18
American Journal of Dermatopathology	112	Dermatologic Surgery	86	Archives of Dermatological Research	26	American Journal of Dermatopathology	71	Journal of Craniofacial Surgery	54	Diagnostics	18
Dermatologic Surgery	102	Journal of the European Academy of Dermatology and Venereology	82	Journal of the American Academy of Dermatology	25	Dermatologic Therapy	67	Asian Pacific Journal of Cancer Prevention	53	Journal of Cutaneous Pathology	17
Dermatologic Therapy	84	Journal of Cutaneous Pathology	69	Biomedical Signal Processing and Control	24	Journal of the American Academy of Dermatology	58	Journal of Cutaneous Pathology	45	Anais Brasileiros De Dermatologia	15
Journal of Cutaneous Pathology	82	Scientific Reports	62	Journal of Cutaneous Pathology	23	Journal of Craniofacial Surgery	57	Journal of the American Academy of Dermatology	43	Melanoma Research	14
British Journal of Dermatology	79	Journal of Craniofacial Surgery	59	Multimedia Tools and Applications	23	Asian Pacific Journal of Cancer Prevention	55	Journal of the European Academy of Dermatology and Venereology	43	Multimedia Tools and Applications	14
Clinical and Experimental Dermatology	64	Asian Pacific Journal of Cancer Prevention	57	IEEE Access	21	Journal of Cosmetic Dermatology	55	Melanoma Research	39	International Journal of Surgery Case Reports	13
Journal of Cosmetic Dermatology	62	British Journal of Dermatology	49	Cancers	20	Journal of Cutaneous Pathology	53	Dermatology	37	Dermatologic Therapy	10

### 3.10. Thematic analysis and keyword co-occurrence patterns

Keyword co-occurrence network analysis reveals substantial thematic diversity and evolving research priorities across skin cancer literature, with notable distinctions between globally collaborative and Middle Eastern-focused datasets (Fig. 7). The visualization employs network topology wherein nodes represent author-selected keywords, node size reflects keyword frequency, connecting lines indicate co-occurrence within individual publications, and distinct color clusters identify thematically related research domains. The analysis reveals substantial thematic diversity and evolving research priorities across skin cancer literature, with notable distinctions between globally collaborative and Middle Eastern-focused datasets (Fig. 7). The visualization demonstrates 4 dataset configurations with varying cluster formations and network densities, reflecting the conceptual organization of research domains. The global collaborative research dataset (5640 documents, Fig. 7A) exhibits 4 thematic clusters with dense interconnectivity (4845 links), dominated by clinical-epidemiological research. Core keywords include “humans” (3452 occurrences), “skin neoplasms” (3367 occurrences), “male” (2973 occurrences), “female” (2898 occurrences), and “adult” (2547 occurrences), reflecting demographic stratification fundamental to epidemiological investigation. Diagnostic entities including “melanoma,” “basal cell carcinoma,” and “squamous cell carcinoma” anchor pathological clusters, while methodological terms such as “controlled study” (1582 occurrences) and “deep learning” (1740 occurrences) indicate integration of advanced computational approaches. Middle Eastern research (Fig. 7C and D) without global collaboration (3869 documents, Fig. 7B) presents 9 distinct clusters with substantially reduced interconnectivity (915 links), suggesting greater thematic fragmentation. Dominant keywords include “skin cancer” (388 occurrences), “melanoma” (324 occurrences), and “basal cell carcinoma” (231 occurrences). Notably, computational methodologies including “deep learning,” “mycosis fungoides,” and “controlled study” demonstrate regional engagement with advanced diagnostic technologies alongside clinical case studies (Fig. 7).

### 3.11. Thematic map

**Figure 8.**
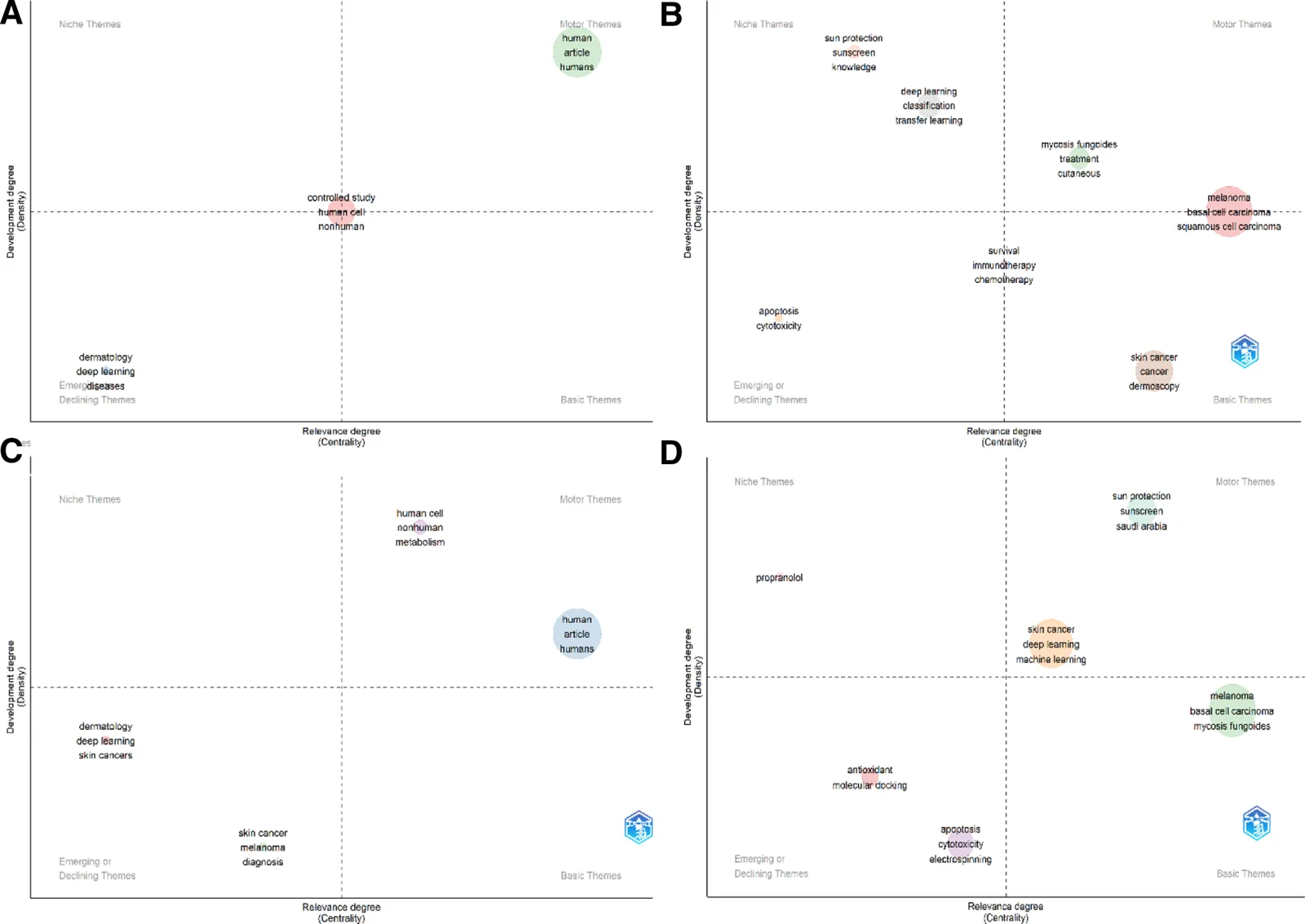
The figure presents 4 thematic maps (A–D) generated using Bibliometrix’s co-word analysis based on author keywords, applying Callon centrality–density algorithm. The *x*-axis (centrality) represents thematic relevance or importance to the research field, while the *y*-axis (density) indicates the degree of internal development or cohesion. Each quadrant identifies different thematic types: motor themes (high relevance and development), basic themes (high relevance but low development), niche themes (high development but limited relevance), and emerging or declining themes (low in both). Bubble size corresponds to keyword frequency, while color intensity reflects thematic strength and conceptual maturity within dermatological and AI-driven skin cancer research. AI = artificial intelligence.

The thematic map (Fig. 8) was developed using Bibliometrix’s co-word analysis based on *author keywords*, applying Callon centrality–density algorithm to evaluate the structural importance (centrality) and developmental strength (density) of each cluster. Four thematic quadrants were identified, each signifying distinct conceptual relevance within the field.

#### 3.11.1. Thematic map (1946–2025; N = 5640; all documents)

Motor themes (upper-right quadrant), including human, article, and humans, exhibit both high density and high centrality, denoting well-developed and influential domains that anchor biomedical and clinical investigations in dermatological research. Basic themes (lower-right quadrant), represented by dermatology, deep learning, and diseases, reflect foundational yet evolving areas that underpin current research frameworks, emphasizing the integration of artificial intelligence with skin disease diagnostics. Niche themes (upper-left quadrant) were absent, indicating limited specialization or isolated topics within the analyzed corpus. Emerging or declining themes (lower-left quadrant), such as controlled study, human cell, and nonhuman, demonstrate moderate connectivity but low density, suggesting early conceptual development or reduced contemporary emphasis. Collectively, the thematic configuration highlights a shift toward human-centered and AI-assisted dermatological research, reflecting methodological advancement and clinical applicability (Fig. 8A).

#### 3.11.2. Thematic map (1946–2025; N = 3869; data-driven studies)

Motor themes (upper-right quadrant), represented by *mycosis fungoides*, *treatment*, and *cutaneous*, display high centrality and density, indicating mature, well-developed research areas that strongly influence the field’s intellectual structure. Basic themes (lower-right quadrant), including *melanoma*, *basal cell carcinoma*, *squamous cell carcinoma*, *skin cancer*, *cancer*, and *dermoscopy*, form the foundational core, representing clinically significant and methodologically stable research lines. Niche themes (upper-left quadrant), such as *sun protection*, *sunscreen*, *knowledge*, *deep learning*, *classification*, and *transfer learning*, exhibit specialized and emerging intersections between artificial intelligence and preventive dermatology. Emerging or declining themes (lower-left quadrant), notably *apoptosis*, *cytotoxicity*, *immunotherapy*, *chemotherapy*, and *survival*, reflect transitional or waning research interests. Overall, the map underscores a shift toward integrative, AI-enhanced diagnostic and therapeutic research in dermatologic oncology (Fig. 8B).

#### 3.11.3. Thematic map (2021–2025; N = 2107; all documents)

Motor themes (upper-right quadrant), including *human cell*, *nonhuman*, and *metabolism*, display both high density and high centrality, representing well-developed, cohesive, and influential research fronts primarily linked to cellular and metabolic processes in dermatological studies. Basic themes (lower-right quadrant), such as *human*, *article*, and *humans*, constitute the structural foundation of the field, providing methodological and conceptual scaffolding across dermatology and biomedical research. Niche themes (upper-left quadrant) were absent, indicating a limited number of specialized, isolated topics. Emerging or declining themes (lower-left quadrant), including *dermatology*, *deep learning*, *skin cancers*, *skin cancer*, *melanoma*, and *diagnosis*, suggest a transition toward computational and diagnostic innovation, emphasizing AI-driven applications in skin cancer detection and classification. Collectively, these findings highlight the field’s ongoing evolution toward integrative, technology-enhanced dermatological research (Fig. 8C).

#### 3.11.4. Thematic map (2021–2025; N = 1260; data-driven studies)

The resulting conceptual landscape comprises 4 quadrants, reflecting different stages of thematic maturity and importance. Motor themes (upper-right quadrant) exhibit both high development and high relevance, encompassing *sun protection*, *sunscreen*, and *Saudi Arabia*, indicating growing regional interest in preventive dermatology and public health awareness. Basic themes (lower-right quadrant), such as *melanoma*, *basal cell carcinoma*, and *mycosis fungoides*, represent foundational research domains central to the field’s conceptual structure. Niche themes (upper-left quadrant), including *propranolol*, are highly specialized and internally cohesive yet less connected to mainstream topics, reflecting emerging pharmacological investigations. Emerging or declining themes (lower-left quadrant), such as *antioxidant* and *molecular docking*, suggest peripheral or evolving research fronts. Central clusters like *skin cancer*, *deep learning*, and *machine learning* bridge technological and clinical dimensions, indicating a methodological shift toward AI-driven diagnostic innovation (Fig. 8C).

A comparative summary of thematic structures across all 4 datasets is presented in Table S1, Supplemental Digital Content 2, highlighting consistent motor themes (e.g., melanoma and humans), evolving basic themes (e.g., AI and dermoscopy), and dataset-specific emerging clusters (e.g., propranolol and antioxidant).

### 3.12. Temporal evolution and emerging research themes

**Figure 9.**
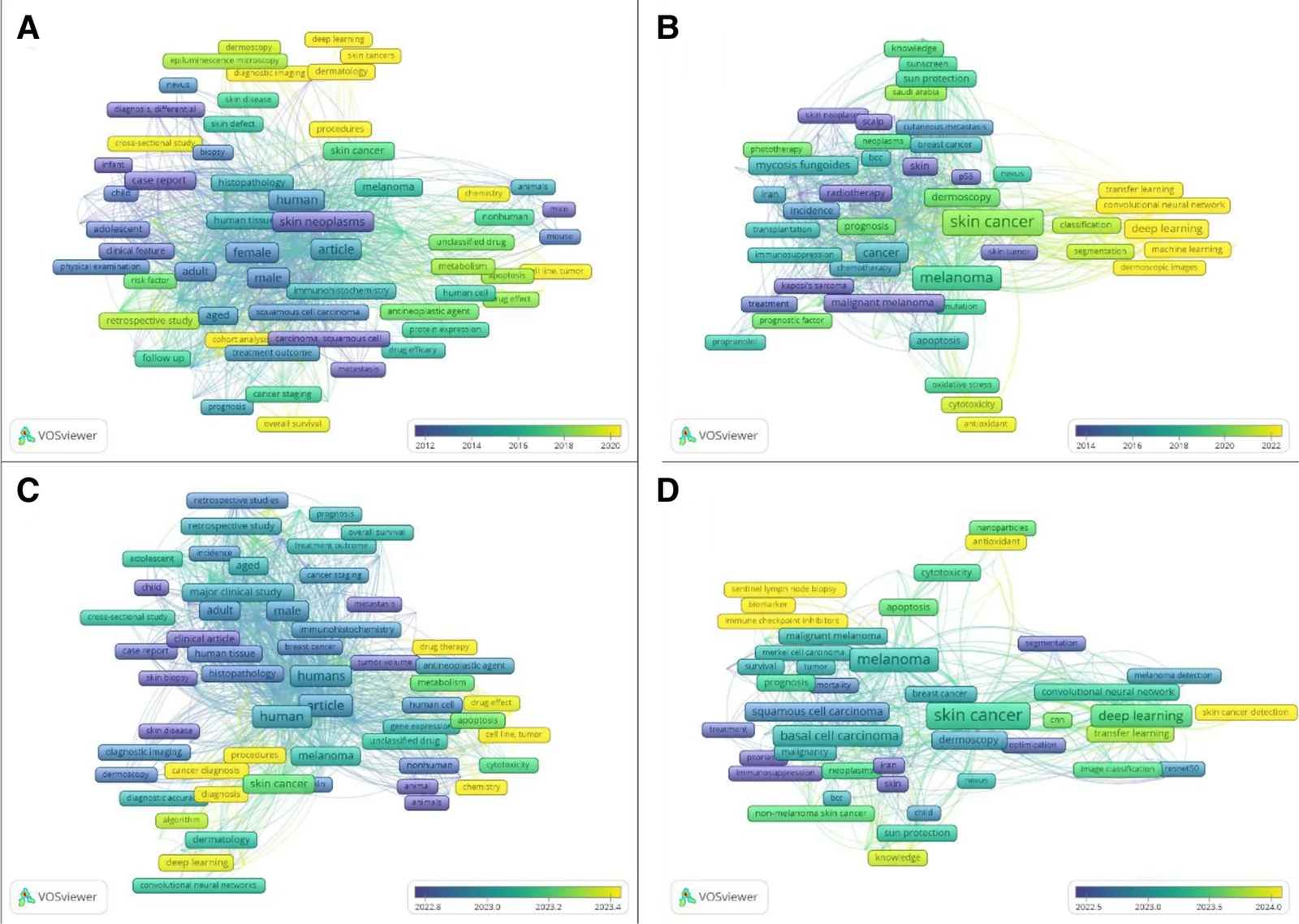
Temporal overlay visualization of emerging research themes in skin cancer literature (VOSviewer analysis). (A) Global collaborative original research (5640 documents); (B) Middle Eastern-focused original research (3869 documents); (C) recent global collaborative research (2021–2025, 2107 documents); and (D) recent Middle Eastern-focused research (2021–2025, 1260 documents). The color gradient represents temporal progression: blue nodes indicate established topics (earlier publications), green nodes represent sustained themes, and yellow nodes denote emerging research areas (most recent publications). Node size reflects keyword frequency; connecting lines indicate co-occurrence patterns. Temporal scales displayed at the bottom indicate average publication years for keywords, enabling identification of declining, stable, and ascending research priorities within the skin cancer research landscape.

Overlay visualization analysis reveals the temporal evolution of research themes and identifies emerging topics within skin cancer scholarship, with distinct patterns observed between globally collaborative and Middle Eastern-focused datasets (Fig. 9). The color gradient – transitioning from blue (older keywords) through green to yellow (emerging topics) – provides temporal context for thematic development, enabling identification of declining, stable, and ascending research priorities. Panel A, representing comprehensive global collaborative research, demonstrates “deep learning,” “skin cancers,” and “overall survival” as prominent emerging themes (yellow nodes), indicating recent intensification of artificial intelligence applications and prognostic research. Traditional clinical-epidemiological terms including “human,” “male,” “female,” and “skin neoplasms” maintain centrality (blue-green nodes), reflecting sustained foundational importance. Panel B, depicting Middle Eastern research, reveals “transfer learning,” “convolutional neural networks,” “deep learning,” “machine learning,” and “segmentation” as dominant emerging themes, suggesting pronounced regional emphasis on computational methodologies for diagnostic enhancement – potentially reflecting strategies to address dermatology workforce limitations through technology-assisted diagnosis. Recent global collaborative research (Panel C, 2021–2025) exhibits emerging focus on “deep learning,” “convolutional neural networks,” “chemistry,” “cytotoxicity,” and “cell line tumor,” indicating convergence of computational approaches with molecular pharmacology. Middle Eastern recent research (Panel D) demonstrates “skin cancer detection,” “melanoma detection,” “deep learning,” “image classification,” and “nanoparticles” as emerging domains, underscoring regional commitment to translational technologies alongside nanotechnology-based therapeutic innovations (Fig. 9).

### 3.13. Cluster analysis (2021–2025; N = 1260; original articles)

**Figure 10.**
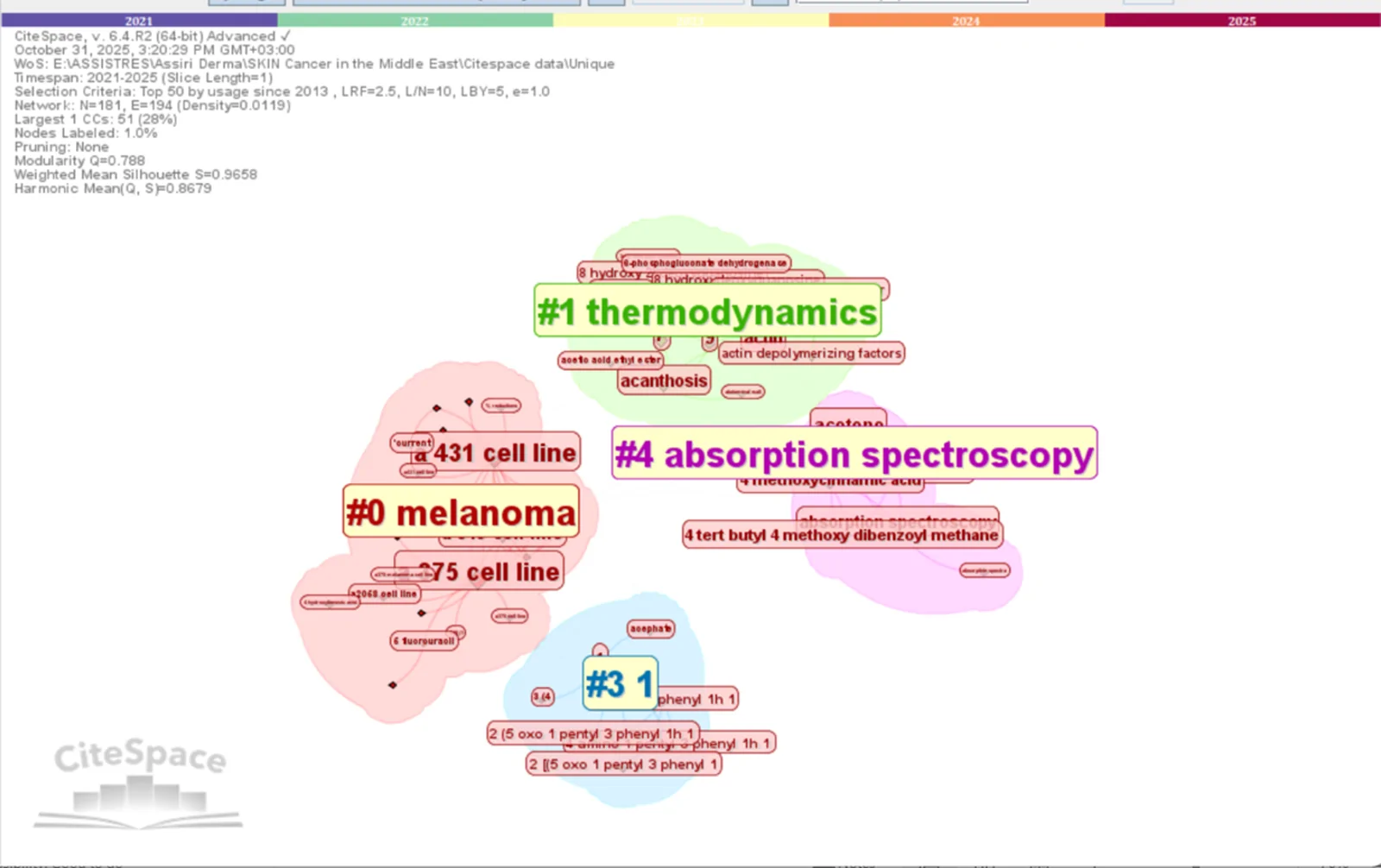
The CiteSpace clustering map (2021–2025) illustrates 4 major thematic clusters in skin cancer research. Node size represents keyword frequency, color indicates cluster identity, and connecting lines show co-occurrence strength, highlighting emerging experimental, molecular, and analytical research fronts in the Middle East.

The CiteSpace-based clustering analysis (Fig. 10) was conducted on the most recent 5-year dataset (2021–2025), comprising 1260 original research articles on skin cancer research in Middle Eastern countries. The resulting network produced 4 major clusters with high modularity (*Q* = 0.788) and strong homogeneity (weighted mean silhouette = 0.9658), indicating a structurally robust and conceptually cohesive landscape. Cluster #0 (“Melanoma”), the largest and most cohesive cluster (n = 24; silhouette = 0.973), centers on melanoma biology and cell line experimentation (notably A375, A431, and A2058), forming the experimental core of regional oncology research. Cluster #1 (“Thermodynamics”) (n = 14; silhouette = 0.966) includes keywords such as thermodynamics, acanthosis, and actin, reflecting a growing interest in biophysical modeling of skin tissue, cytoskeletal dynamics, and early neoplastic changes. Cluster #3 (“Mechanistic Toxicology”) (n = 7; silhouette = 0.958) contains terms related to chemical derivatives and toxicity profiling, indicative of mechanistic investigations into skin-relevant anticancer agents, nanocarriers, and oxidative stress modulators. Cluster #4 (“Absorption Spectroscopy”) (n = 6; silhouette = 0.945) emphasizes spectroscopic and photochemical approaches, including UV-visible and near-infrared techniques applied to the characterization of sunscreens, plant-based photoprotective agents, and nanoparticle formulations. This cluster was especially active in studies from Saudi Arabia and Iran. Collectively, these clusters reflect a thematic shift toward molecular experimentation, translational toxicology, and novel screening platforms in regional skin cancer research.

## 4. Discussion

This study provides the 1st comprehensive informetric evaluation of skin cancer research in Middle Eastern countries, revealing a rapidly evolving landscape characterized by increased scientific output and growing integration of artificial intelligence and molecular methodologies. The findings highlight distinct thematic structures driven by regional priorities, emphasizing melanoma, dermoscopy, and preventive dermatology as central research foci. These results address the initial hypothesis that skin cancer research in the region is transitioning toward technologically enhanced, translational, and collaborative domains aligned with global scientific trends.

The temporal and spatial analysis of skin cancer research from 1946 to 2025 reveals a significant global rise in scholarly output, with the Middle East exhibiting notable yet uneven growth. While global publication volume surged exponentially post-2000, driven by technological advances and increasing cancer incidence,^[24,25]^ Middle Eastern contributions – once minimal – expanded markedly, especially after 2010.^[26]^ Turkey, Iran, and Egypt dominate regional output, collectively accounting for the majority of publications. Recent trends suggest Iran may surpass Turkey, signaling shifts in regional research leadership. However, smaller nations like Bahrain and Yemen remain underrepresented, highlighting disparities in research infrastructure and funding.^[27]^ Quality-filtered datasets reveal that over 70% of Turkish and Iranian publications are peer-reviewed, underscoring methodological rigor. The growing integration of artificial intelligence, nanotechnology, and molecular science into regional research reflects a shift toward advanced dermatologic oncology.^[28,29]^ Despite progress, collaboration networks remain weak, particularly intra-regionally. Strengthening partnerships, aligning research with epidemiological needs, and investing in translational capacity will be vital for equitable advancement. Overall, Middle Eastern research is maturing, offering essential insights into skin cancer while contributing to the broader global scientific landscape.

The interpretation of GDP versus incidence data reveals striking disparities, particularly in Gulf Cooperation Council countries where high economic development coincides with relatively low reported NMSC incidence. While the manuscript previously emphasized underreporting due to gaps in registry completeness, this interpretation may be overly deterministic. Alternative explanations – such as prevalent indoor lifestyles, cultural clothing practices that reduce UV exposure, and sun-avoidance behaviors – offer equally plausible epidemiological justifications for genuinely lower incidence in these nations (e.g., Qatar, UAE, and Saudi Arabia). These factors warrant careful consideration in conjunction with structural limitations in cancer surveillance systems. Therefore, while underreporting remains a valid concern – especially in light of inconsistent registry coverage and limited open-access datasets – it should be framed as 1 of multiple contributing variables. Future work should investigate the relative impact of behavioral, cultural, and infrastructural factors through integrated epidemiological and health system assessments to draw more definitive conclusions about skin cancer incidence patterns across the region.

This comprehensive survey expands on the citation impact analysis presented in the results, drawing from bibliometric data to contextualize the observed heterogeneity across countries in skin cancer research. The VOSviewer density visualizations (Fig. 5) underscore marked distinctions in citation patterns between globally collaborative datasets and those focused on the Middle East, revealing a “citation premium” associated with international partnerships. This premium manifests as elevated per-document citations and denser network integrations for nations engaging in cross-border collaborations, a phenomenon well-documented in medical research literature. The findings highlight a citation premium from global collaborations in skin cancer research, aligning with broader trends where international partnerships enhance impact, as seen in palliative care studies showing higher citations for collaborative works.^[30]^ Israel’s high efficiency (45.4 citations/document) mirrors its integration in global networks, consistent with bibliometric analyses of melanoma in Arab countries, where Egypt and Saudi Arabia lead publications but lag in per-capita impact.^[26]^ Turkey’s regional dominance without collaboration reflects emerging Middle Eastern trends, akin to Iran’s focus in cancer nanotechnology.^[31]^ Globally, USA and European hubs dominate, paralleling Photodynamic therapy and AI skin cancer research patterns.^[32,33]^ Implications include fostering collaborations to elevate Middle Eastern research visibility, potentially accelerating nanotechnology innovations in skin cancer therapy.^[16]^

The network analysis highlights a collaboration premium in global skin cancer research, with the United States as the central hub (link strength: 2549), echoing dominance in AI-driven skin cancer studies where it leads publications and centrality.^[32]^ Israel’s robust connectivity (1854) aligns with its integration in international oncology networks, contrasting Middle Eastern countries’ lower strengths, similar to Arab oncology trials showing minimal joint non-Arab participation (12.5%).^[14]^ Saudi Arabia’s regional leadership (76) reflects emerging intra-MENA ties, as seen in UAE cancer research with 40% international co-authorship but limited South–South links.^[13]^ Recent trends (2021–2025) indicate growing regional networks, paralleling Saudi cancer bibliometrics with strong Egypt-India-US partnerships.^[28]^ In fragile MENA settings, low intra-regional collaboration underscores infrastructure gaps, akin to African cancer research’s high North–South dependence.^[34,35]^ Implications include promoting South–South frameworks to enhance capacity, potentially accelerating nanotechnology advancements in skin cancer via shared regional hubs.

The thematic evolution of skin cancer research in the Middle East is profoundly shaped by the interplay of dataset scope, collaboration patterns, and temporal depth. Longitudinal, globally collaborative datasets foster broad, methodologically integrated research agendas anchored in foundational biomedical themes.^[30,34,36]^ In contrast, shorter, region-specific, and non-collaborative datasets emphasize emerging or locally relevant topics, often with limited conceptual cohesion. This divergence underscores the critical role of international engagement and extended research horizons in cultivating mature, high-impact thematic structures. Strengthening global partnerships and sustaining long-term research efforts will be essential to advance the thematic maturity and global relevance of Middle Eastern dermatologic oncology research.^[13,28]^ The 4 thematic maps (Fig. 8A–D) collectively illustrate how data type, global collaboration, and study duration shape the thematic evolution of *skin cancer research in Middle Eastern countries*. In the comprehensive global dataset (1946–2025; All Documents, Fig. 8A), themes are broad and human-centered, dominated by general biomedical terms (*human*, *article*, and *humans*), reflecting globally integrated, multidisciplinary dermatology research. When limited to data-driven studies (1946–2025; Fig. 8B), the thematic structure becomes more specialized, emphasizing *melanoma*, *basal cell carcinoma*, *squamous cell carcinoma*, and *dermoscopy*, alongside emerging intersections with *AI* and *deep learning*, indicating strong methodological refinement and technological adaptation. The recent dataset (2021–2025; All Documents, Fig. 8C) reveals a narrowing of focus toward *metabolism* and *human cell* processes, showing higher conceptual maturity but reduced thematic diversity – likely a result of shorter time frames and concentrated research efforts. Conversely, the recent data-driven subset (2021–2025; Fig. 8D) highlights localized, applied research (*sun protection*, *sunscreen*, and *Saudi Arabia*), suggesting an increasing regional engagement in public health–oriented and AI-enhanced dermatology. Overall, longer time spans capture broader, foundational trends, while shorter and region-specific datasets reveal emerging public health and computational themes. The presence or absence of international collaboration markedly affects thematic depth – globally coauthored works yield more cohesive and influential clusters, whereas nationally driven studies tend toward narrower, context-specific, or emerging topics.^[13,28]^

For skin cancer research, these findings imply that global partnerships are essential for thematic richness, potentially accelerating AI-driven innovations like SkinGPT-4 for diagnosing melanoma and basal cell carcinoma in high-UV regions.^[37,38]^ In the Middle East, where output lags (e.g., Arab nations 1.52% of global cancer publications), fostering intra-regional networks – leveraging hubs like Saudi Arabia (regional leader with Egypt, USA ties) – could address gaps in fragile settings (e.g., Iraq’s 82.7% single-country papers).^[15,34]^ Public health themes signal opportunities for interventions, given awareness deficits (e.g., 16.69% unaware of sun-cancer link in Saudi students), to reduce incidences tied to socioeconomic factors.^[39,40]^ The research community should prioritize policy for funding South–South collaborations, integrating AI curricula, and translational studies to align with global trends (e.g., ensemble learning in diagnostics).^[32]^ Challenges include political instability and resource disparities, but implications extend to equitable health outcomes, with enhanced visibility fostering precision oncology in underrepresented areas.

The trend analysis (Fig. 9) indicates that deep learning has emerged as a prominent theme in skin cancer research within the Middle East, as evidenced by a surge in innovative studies from regional scholars in 2025. For instance, researchers from Saudi Arabia and Turkey have developed advanced convolutional neural network and vision transformer hybrids to enhance lesion segmentation and classification accuracy on benchmark datasets like international skin imaging collaboration and HAM10000.^[41–43]^ These efforts address diagnostic challenges such as subtle visual differences between benign and malignant lesions, incorporating dual encoders for efficient feature extraction and achieving up to 97.1% accuracy.^[41,42]^ Similarly, Iraqi and Saudi teams have introduced frameworks like Skin-DeepNet and dual-stream models that integrate preprocessing, attention mechanisms, and histopathological features, yielding near-perfect segmentation (intersection over union up to 99.93%) and classification rates exceeding 99%.^[44,45]^ Turkish contributions emphasize optimized MobileNetV2 with memetic algorithms for resource-efficient prediction, attaining 98.48% accuracy with interpretability via gradient-weighted class activation mapping.^[46]^ Beyond the Middle Eastern core, collaborative works with South Asian affiliates, such as hybrid SkinEHDLF (an advanced hybrid deep learning framework built for precise skin cancer classification) and focal modulation network, demonstrate adaptive fusion and focal modulation for multi-class tasks, reducing false positives by 28% and achieving Jaccard indices up to 89.60%.^[47,48]^ Privacy-focused federated learning models from Pakistani groups ensure data security while maintaining high performance (93.48% accuracy), highlighting ethical AI integration.^[49,50]^ This proliferation underscores the region’s growing investment in AI-driven oncology, fostering global collaborations and potentially bridging diagnostic gaps in underserved areas.

While the rise in publications on AI, dermoscopy, and molecular techniques reflects promising thematic shifts, the practical integration of these technologies into clinical dermatology across the Middle East remains limited. Challenges include a lack of standardized clinical guidelines, regulatory frameworks for digital health, and limited infrastructure for AI deployment in dermatologic practice. Thus, the observed publication surge likely signals early academic interest rather than widespread clinical transformation. Furthermore, although linguistic, financial, and geopolitical barriers impede collaboration, regional hubs such as Saudi Arabia, Egypt, and Turkey – by virtue of their institutional capacity, research output, and relative political influence – are well positioned to catalyze South–South cooperation. These countries could lead targeted consortia, training exchanges, and shared research funding programs, helping bridge capacity gaps in lower-resource nations and fostering a more integrated regional research ecosystem.

This study has several limitations. First, it relied exclusively on Scopus-indexed publications, which, while offering broad international coverage, may underrepresent regional, non-English, or locally disseminated literature. This could result in the exclusion of relevant research from Middle Eastern countries with lower indexing rates. Cross-verification with other databases – such as Web of Science, PubMed, and regional repositories like the Index Medicus for the Eastern Mediterranean Region – is recommended in future research. Second, bibliometric indicators assess publication volume and network patterns but do not directly measure methodological quality, translational relevance, or clinical impact. Third, country affiliations derived from metadata may incompletely reflect multinational collaborations, particularly when institutions from multiple nations contribute to a single study. Finally, while this study applied quantitative bibliometric techniques, future work should incorporate qualitative content analysis, explore citation network dynamics, and assess policy and funding contexts to provide a more holistic understanding of research trends and structural barriers in the region.

## 5. Conclusions

This comprehensive bibliometric investigation provides an in-depth view of the intellectual structure, collaborative dynamics, and thematic progression of skin cancer research in the Middle East within a global framework. The analysis highlights a 7.38% annual growth rate, significant contributions from Turkey, Iran, and Egypt, and a thematic evolution from clinical epidemiology toward AI-driven diagnostics, dermoscopy, and molecular experimentation. Despite these advances, the region continues to underperform in international collaboration (2.74%) and citation impact relative to global benchmarks. Clustering analyses revealed emerging experimental domains, including mechanistic toxicology and absorption spectroscopy, suggesting growing interest in biophysical modeling, nanoparticle-based therapeutics, and photoprotective strategies. These developments may inform future translational applications in chemoprevention, targeted therapy, and personalized dermatologic care. To realize this potential, regional stakeholders must strengthen research infrastructure, establish unified cancer registries, and expand investment in computational and molecular innovation. This study lays a data-driven foundation for policy alignment, research capacity building, and clinical integration – critical steps for advancing dermatologic oncology across the Middle East.

## Author contributions

**Conceptualization:** Ahmad Assiri.

**Data curation:** Ahmad Assiri.

**Formal analysis:** Ahmad Assiri.

**Funding acquisition:** Ahmad Assiri.

**Investigation:** Ahmad Assiri

**Methodology:** Ahmad Assiri.

**Project administration:** Ahmad Assiri.

**Resources:** Ahmad Assiri.

**Software:** Ahmad Assiri.

**Supervision:** Ahmad Assiri.

**Validation:** Ahmad Assiri.

**Visualization:** Ahmad Assiri.

**Writing – original draft:** Ahmad Assiri.

**Writing – review & editing:** Ahmad Assiri.




