# Supplementary material for: The intellectual structure and thematic evolution of skin cancer research in the Middle East: A data-driven bibliometric exploration of global integration and emerging fronts (1946–2025)
Source: Medicine (Baltimore). 2026 Aug 28;105(35):e50460. doi: 10.1097/MD.0000000000050460 (PMC13529154; doi:10.1097/MD.0000000000050460)
Supplement: Supplementary file 1 [file medi-105-e50460-s001.docx]

TITLE-ABS-KEY ( "skin cancer"  OR  "cutaneous cancer"  OR  "cutaneous malignancy"  OR  "skin carcinoma"  OR  "skin malignancy"  OR  "cutaneous neoplasm"  OR  "cutaneous neoplasms"  OR  "skin neoplasm"  OR  "skin neoplasms" )

179,158 document results, Global literature

1850 – 2025

( TITLE-ABS-KEY ( "skin cancer" OR "cutaneous cancer" OR "cutaneous malignancy" OR "skin carcinoma" OR "skin malignancy" OR "cutaneous neoplasm" OR "cutaneous neoplasms" OR "skin neoplasm" OR "skin neoplasms" ) AND AFFILCOUNTRY ( "Bahrain" OR "Cyprus" OR "Egypt" OR "Iran" OR "Iraq" OR "Israel" OR "Jordan" OR "Kuwait" OR "Lebanon" OR "Oman" OR "Palestine" OR "Qatar" OR "Saudi Arabia" OR "Syria" OR "Turkey" OR "United Arab Emirates" OR "UAE" OR "Yemen" ) )))

7,932 documents found, Middle East

1946 – 2025

( TITLE-ABS-KEY ( "skin cancer" OR "cutaneous cancer" OR "cutaneous malignancy" OR "skin carcinoma" OR "skin malignancy" OR "cutaneous neoplasm" OR "cutaneous neoplasms" OR "skin neoplasm" OR "skin neoplasms" ) AND AFFILCOUNTRY ( "Bahrain" OR "Cyprus" OR "Egypt" OR "Iran" OR "Iraq" OR "Israel" OR "Jordan" OR "Kuwait" OR "Lebanon" OR "Oman" OR "Palestine" OR "Qatar" OR "Saudi Arabia" OR "Syria" OR "Turkey" OR "United Arab Emirates" OR "UAE" OR "Yemen" ) ) AND ( EXCLUDE ( PUBYEAR , 2026 ) ) AND ( LIMIT-TO ( DOCTYPE , "ar" ) ) AND ( LIMIT-TO ( LANGUAGE , "English" ) )

5,640 document results, Middle East

1946 – 2025

( TITLE-ABS-KEY ( "skin cancer"  OR  "cutaneous cancer"  OR  "cutaneous malignancy"  OR  "skin carcinoma"  OR  "skin malignancy"  OR  "cutaneous neoplasm"  OR  "cutaneous neoplasms"  OR  "skin neoplasm"  OR  "skin neoplasms" )  AND  AFFILCOUNTRY ( "Bahrain"  OR  "Cyprus"  OR  "Egypt"  OR  "Iran"  OR  "Iraq"  OR  "Israel"  OR  "Jordan"  OR  "Kuwait"  OR  "Lebanon"  OR  "Oman"  OR  "Palestine"  OR  "Qatar"  OR  "Saudi Arabia"  OR  "Syria"  OR  "Turkey"  OR  "United Arab Emirates"  OR  "UAE"  OR  "Yemen" ) )  AND  ( LIMIT-TO ( PUBYEAR ,  2021 )  OR  LIMIT-TO ( PUBYEAR ,  2022 )  OR  LIMIT-TO ( PUBYEAR ,  2023 )  OR  LIMIT-TO ( PUBYEAR ,  2024 )  OR  LIMIT-TO ( PUBYEAR ,  2025 )  OR  EXCLUDE ( PUBYEAR ,  2026 ) )  AND  ( LIMIT-TO ( DOCTYPE ,  "ar" ) )  AND  ( LIMIT-TO ( LANGUAGE ,  "English" ) )

2,107 document results, Middle East

2021 - 2025
