# Supplementary material for: The intellectual structure and thematic evolution of skin cancer research in the Middle East: A data-driven bibliometric exploration of global integration and emerging fronts (1946–2025)
Source: Medicine (Baltimore). 2026 Aug 28;105(35):e50460. doi: 10.1097/MD.0000000000050460 (PMC13529154; doi:10.1097/MD.0000000000050460)
Supplement: Supplementary file 2 [file medi-105-e50460-s002.docx]

Supplementary Table S1. Comparison of Dominant Themes in Thematic Map Quadrants Across Dataset Configurations

| Thematic Quadrant | 1946–2025 (All Docs) | 1946–2025 (Data-Driven Studies) | 2021–2025 (All Docs) | 2021–2025 (Data-Driven Studies) |
| --- | --- | --- | --- | --- |
| Motor Themes | Humans, Article, Humans | Mycosis fungoides, Treatment, Cutaneous | Human cell, Nonhuman, Metabolism | Sun protection, Sunscreen, Saudi Arabia |
| Basic Themes | Dermatology, Deep learning, Diseases | Melanoma, BCC, SCC, Skin cancer, Cancer, Dermoscopy | Human, Article, Humans | Melanoma, BCC, Mycosis fungoides |
| Niche Themes | None | Sun protection, Sunscreen, Knowledge, Deep learning | None | Propranolol |
| Emerging/Declining | Controlled study, Human cell, Nonhuman | Apoptosis, Cytotoxicity, Immunotherapy, Chemotherapy | Dermatology, Deep learning, Melanoma, Diagnosis | Antioxidant, Molecular docking |
